# Genetic diagnosis and clinical characteristics analysis of cardiospondylocarpofacial syndrome in a Chinese family

**DOI:** 10.3389/fped.2025.1651803

**Published:** 2025-08-20

**Authors:** Qi Yang, Qiang Zhang, Sheng Yi, Wurui Li, Xunzhao Zhou, Shujie Zhang, Shang Yi, Qinle Zhang, Jingsi Luo

**Affiliations:** ^1^ Guangxi Key Laboratory of Birth Defects Research and Prevention, Guangxi Key Laboratory of Reproductive Health and Birth Defects Prevention, Maternal and Child Health Hospital of Guangxi Zhuang Autonomous Region, Nanning, China; ^2^ Department of Genetic and Metabolic Central Laboratory, Maternal and Child Health Hospital of Guangxi Zhuang Autonomous Region, Nanning, China; ^3^ Guangxi Clinical Research Center for Birth Defects, Maternal and Child Health Hospital of Guangxi Zhuang Autonomous Region, Nanning, China; ^4^ Paediatric Cardiovascular Medicine, Maternal and Child Health Hospital of Guangxi Zhuang Autonomous Region, Nanning, China; ^5^ Guangxi Clinical Research Center for Pediatric Diseases, Maternal and Child Health Hospital of Guangxi Zhuang Autonomous Region, Nanning, China

**Keywords:** cardiospondylocarpofacial syndrome, *MAP3K7*, novel *de novo* variant, whole-exome sequencing, new complications

## Abstract

Cardiospondylocarpofacial syndrome (CSCFS) is an extremely rare autosomal dominant disorder resulting from variant in the *MAP3K7* gene, which encodes the transforming growth factor-β-activated kinase 1 (TAK1). Only 26 cases of CSCFS have been reported worldwide. The main manifestations are growth retardation, hypotonia, dysmorphic facial features, skeletal and limb abnormalities, cardiac septal defects with valve dysplasia, cardiomyopathy, and deafness with inner ear malformations. In this study, we recruited an unrelated Chinese family with a patient diagnosed with CSCFS. Whole exome sequencing revealed a novel heterozygous variant, c.142G > A[p. (Gly48Arg)], in the *MAP3K7* gene. The variant was confirmed by Sanger sequencing to be absent in other family members and is *de novo*. The patient described here has a similar dysmorphology profile to that associated with CSCFS. Compared with reported cases of CSCFS, our patient presented with new complications of short tongue tie, brain abnormalities including asymmetrical cerebral hemispheres with widening of the right frontotemporal exoptic hiatus, intestinal obstruction and intussusception. In addition, scoliosis, vertebral abnormalities, carpal/tarsal fusion, pectus excavatum, and cervical spine fusion were not found in our patient. The molecular diagnosis in this patient extends the known genetic spectrum of CSCFS. Furthermore, the specific manifestations in this case offer valuable additional clinical details regarding the syndrome.

## Introduction

Mitogen-activated protein kinase kinase kinase 7 (MAP3K7), encodes the transforming growth factor-β-activated kinase 1 (TAK1) widely expressed in most tissues and is critical for embryonic development (1). TAK1 is a highly conserved serine-threonine kinase that forms a complex with its associated binding proteins TAB1, TAB2, and TAB3 and modulates several downstream effectors, including c-Jun N-terminal kinases (JNKs), extracellular signal-regulated kinases (ERKs), p38 MAPK, and nuclear factor-κB (NF-κB) (2–6). These pathways influence a wide range of cellular processes, such as cell growth and differentiation, immune function, stress responses, and apoptosis. Variants in the *MAP3K7* gene have been linked to two distinct disorders: frontometaphyseal dysplasia type 2 (FMD2) and cardiospondylocarpofacial syndrome (CSCF) (7, 8). FMD2 is a progressive skeletal dysplasia that shares overlapping symptoms with FMD1, which is caused by *FLNA* gene mutations (7, 9). Their common clinical features mainly include cranial and long bone sclerosis, prominent supraorbital ridges, and finger abnormalities. In addition to skeletal manifestations, some patients may present with extra-skeletal symptoms such as hearing loss, urogenital problems, and joint contractures (7, 9). Cardiospondylocarpofacial (CSCF) syndrome is a multisystem congenital disorder, manifests through growth retardation, hypotonia, dysmorphic facial features, brachydactyly with carpal/tarsal synostosis, posterior cervical vertebral fusion that congenital heart defects, deafness with inner ear malformations, and other congenital abnormalities (8). Crucially, *MAP3K7* mutations in CSCFS are characterized by loss-of-function (LOF) effects, which contrasts mechanistically with the gain-of-function (GOF) variants that cause Frontometaphyseal Dysplasia Type 2 (FMD2) (7, 10). *MAP3K7* gene loss-of-function mutations further cause CSCFS through the TGF-β pathway (8). In addition, CSCFS exhibits clinical overlap with Noonan syndrome, but it has not been possible to determine whether it belongs to the RAS disease group (8, 11, 12). To date, only 18 *MAP3K7* gene variants have been reported in patients with CSCFS (8, 10, 12–17). It is not entirely clear how different variants in the same *MAP3K7* gene lead to different clinical phenotypes. More reports on *MAP3K7* variants and their respective phenotypes will help to better understand the disease and explore the relationship between genotype and phenotype.

**Figure 1:**
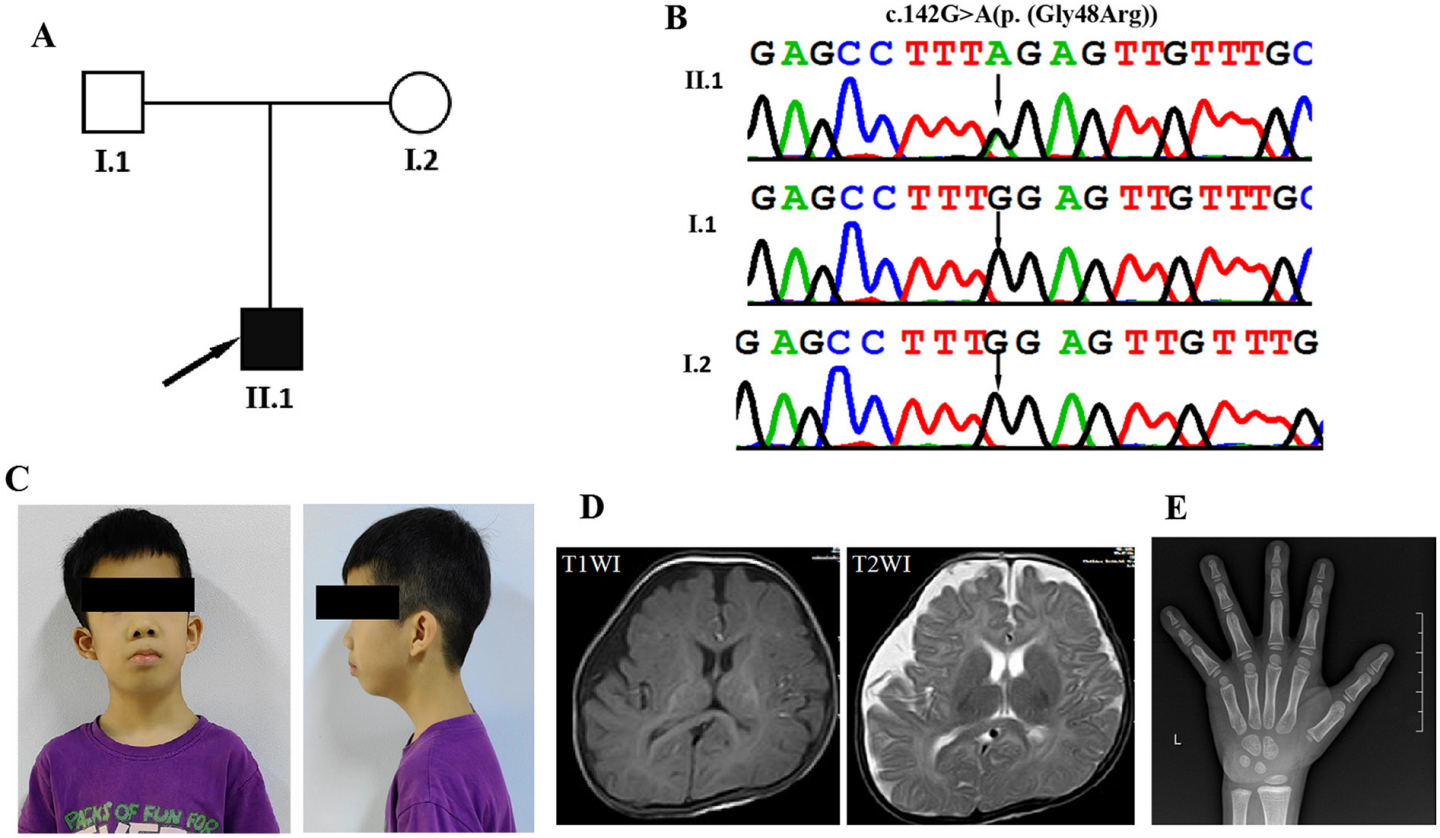
Clinical and genetic features. **(A)** The family pedigree demonstrates that the proband has cardiospondylocarpofacial syndrome. **(B)** DNA sequence chromatograms from Sanger sequencing of MAP3K7, showing a *de novo* heterozygous variant c.142G > A[p. (Gly48Arg)] in the proband (II-1). Further Sanger sequencing indicated that the variant was not identified in his parents (I:1 and I:2). **(C)** Facial appearance of the proband (II-1) at the age of 7 years and 3 months, showing a long face, full cheeks, low-set and prominent ears, entropion, epiblepharon, anteverted nares, long philtrum, ptosis, down-slanting palpebral fissures, and short lingual frenulum. **(D)** Axial slices of T1-weighted images (T1WI) and T2WI acquired at 5 months in the proband (II-1) show bilateral hemispheric asymmetry and widening of the right frontotemporal extracerebral space. **(E)** In the wrist x-ray taken at the age of 6, the bone age is compatible with the age of 5 years.

In this study, we identified a novel *de novo* heterozygous missense variant in *MAP3K7* using whole exome sequencing (WES) in a Chinese patient (Figures 1A,B). The clinical phenotypes of the patient are mostly consistent with those previously reported. We have also uncovered several novel clinical features associated with the *MAP3K7* variant that enrich our understanding of the phenotypes.

## Materials and methods

### Patients

A Chinese family with autism and speech delay was referred to the Pediatric Rehabilitation Department of Guangxi Maternal and Child Health Hospital for genetic evaluation (Figure 1A). The study protocol was approved by the Institutional Review Board and Ethics Committee of the Guangxi Maternal and Child Health Hospital and conducted in accordance with the principles of the Declaration of Helsinki. Written informed consent was obtained from the parents of the affected individuals for the publication of their clinical data and photographs.

### Whole exome sequencing and sanger sequencing

Peripheral blood lymphocytes (2 ml) were collected from the patient and his family members, and genomic DNA was extracted using the Lab-Aid DNA kit (Zeshan Biotechnology Co., Ltd., Xiamen, China) according to the manufacturer's protocol. Whole-exome sequencing was conducted with an Agilent SureSelect V5 enrichment capture kit (Agilent Technologies, Santa Clara, CA, USA), after which 100 bp paired-end sequencing was performed on the Illumina HiSeq 2,000 platform (Illumina, San Diego, CA, USA). The sequencing reads were aligned to the hg19/GRCh37 human reference genome using the Genome Analysis Toolkit (GATK, version 3.4; Broad Institute, Cambridge, MA, USA). Variant calling and annotation were done via TGex software (http://tgex.genecards.org/), with a focus on variants with a minor allele frequency (MAF) of ≤0.001 in public databases (1,000 Genomes Project, Exome Sequencing Project, and Exome Aggregation Consortium). The functional impact of candidate variants was predicted using in silico tools (REVEL, PolyPhen2, SIFT, LRT, Mutation Assessor, CADD, and MutationTaster). The Swiss-Model server (https://swissmodel.expasy.org/) was used to generate the 3D protein structures of *MAP3K7*. The pathogenicity of the candidate variants was assessed in line with the guidelines of the American College of Medical Genetics and Genomics and the ClinGen Sequence Variant Interpretation (SVI) Working Group (18).

## Result

The proband (II:1) is a boy of 7 years and 3 months of age with healthy, unrelated Chinese parents (Figure 1C). He was admitted to the Pediatric Rehabilitation Department of Guangxi Maternal and Child Health Hospital for developmental delay and failure to thrive when he was 5 months old. He was delivered by Caesarean section in the 40th week of pregnancy due to foetal macrosomia. His birth weight was 4,000 g (>90 percentile), birth length 47.5 cm (<25 percentile), and head circumference 40 cm (>97 percentile). Apgar scores were 10, 10 and 10 at 1, 5 and 10 min, respectively. At the age of 4 months and 22 days, the Gesell Developmental Diagnostic Scale was used to assess his Developmental Quotient (DQ, DQ < 70 as low score) (gross motor 29, fine motor 63, adaptive 60, language 41 and personal-social 49). Brain Magnetic resonance imaging at 5 months of age showed bilateral hemispheric asymmetry and widening of the right frontotemporal extracerebral space (Figure 1D). He had hypotonia and delays in developmental milestones. He was able to control his head at 5 months, roll over at 7 months, sit up at 9 months, crawl at 10 months, pull himself to stand at 17 months, and walk at 22 months. He began to speak single words at 18 months and to form sentences at 2 years. A hand x-ray taken at the age of 6 years showed mildly delayed bone age (Figure 1E). Magnetic resonance imaging of the spine showed normal cervical, spinal, thoracic and caudal spines. At the age of 5 years and 5 months, he was diagnosed with dilated cardiomyopathy. Echocardiography showed mild left ventricular dilatation, a mild decrease in left ventricular shortening (FS) (25.6%), pulmonary hypertension, and mild tricuspid and mitral regurgitation. His 24 h ambulatory electrocardiogram monitoring revealed sinus rhythm with an average heart rate of 85 bpm, along with junctional tachycardia, atrioventricular dissociation, occasional atrial premature contractions (APCs), and ventricular premature contractions (VPCs). At the age of 4 years and 11 months, the patient underwent intestinal resection-and-ostomy due to intestinal obstruction and intussusception. Subsequently, he also underwent a descending testicular fixation for cryptorchidism. In addition, the patient has been diagnosed with bilateral secretory otitis media and sinusitis. He was also diagnosed with conductive hearing impairment with an average hearing threshold of 52.6 dB. His last physical examination was at the age of 7 years and 3 months and revealed short stature (108 cm, <-3 SD), brachydactyly, bilateral flat feet, hypermobility of joints throughout the body, atrophic abdominal surgery scars, cryptorchidism, and a small penis. He was diagnosed with a mild intellectual disability. When assessed at the age of 7 years and 3 months using the Wechsler Intelligence Scale for Children, his Full Scale IQ score was determined to be 71. His dysmorphic features include a long face, full cheeks, low-set and prominent ears, entropion, epiblepharon, anteverted nares, long philtrum, ptosis, down-slanting palpebral fissures, and short lingual frenulum (Figure 1C).

### Genome analysis

Whole exome sequencing was performed to determine the genetic basis of the patient's multiple malformations. A total of 5.3 Gb of data was obtained based on whole-exome sequencing, with 99.5% coverage of the target region and 99.0% of the targets covered over 20X. A total of 870 single nucleotide variants (SNVs) and insertion/deletion (indel) variants were selected in the coding region and splice site (10 bp from the splice junction), following the filtering out of synonymous variants and those with a minor allele frequency (MAF) of greater than 1%, from both local and public databases. After further excluding benign variants and likely benign variants (including missense, synonymous, and splicing variants predicted to be harmless by in silico tools), a total of 435 variants remained. Using TGex software (LifeMap Sciences, USA), six gene variants (*SETBP1*, *YARS1*, *MAP3K7*, *COG1*, *ABCA2*, and *CLN8*) associated with the patient's phenotype were identified from genes listed in OMIM. Following comprehensive screening based on inheritance pattern, variant origin, and pathogenicity, a novel *de novo* missense variant c.142G > A[p. (Gly48Arg)] in the *MAP3K7* gene emerged as the most plausible candidate for the patient's condition. This missense variant was classified as likely pathogenic according to the ACMG/AMP guidelines (Table 1) (18). No pathogenic or likely pathogenic copy number variations (CNVs) were detected in the patient's whole-exome sequencing (WES) data using XHMM software with deep-coverage analysis.

**Table 1:** Predicted pathogenicity of *de novo MAP3K7* variant.

Gene	Variant (NM_145,331.2)	Inheritance	LRT	Mutationtaster	Revel	PolyPhen-2	SIFT	CADD	ACMG/AMP
*MAP3K7*	c.142G > A[p. (Gly48Arg)]	DNV	D	D	0.96	D	D	32.0	LP(PS2 + PM2 + PM5 + PP3)

DNV, *de novo* variant; D, deleterious or damaging; D, damaging; LP, likely pathogenic.

## Discussion

Cardiospondylocarpofacial syndrome (CSCFS) is a very rare autosomal dominant disorder. It was first described in 1966 and defined in 2010, followed by the identification of the *MAP3K7* gene as its etiology in 2016 (8, 19, 20). To date, a total of 26 patients with CSCFS have since been reported (8, 10, 12–17). The clinical presentation of these patients with *MAP3K7* variant includes facial dysmorphism, growth retardation, brachydactyly with carpal/tarsal synostosis, posterior cervical vertebral fusion, congenital heart defects and deafness with inner ear malformations. In this study, we performed WES analysis and identified a heterozygous missense variant in the *MAP3K7* gene in a Chinese patient with CSCFS.

**Figure 2:**
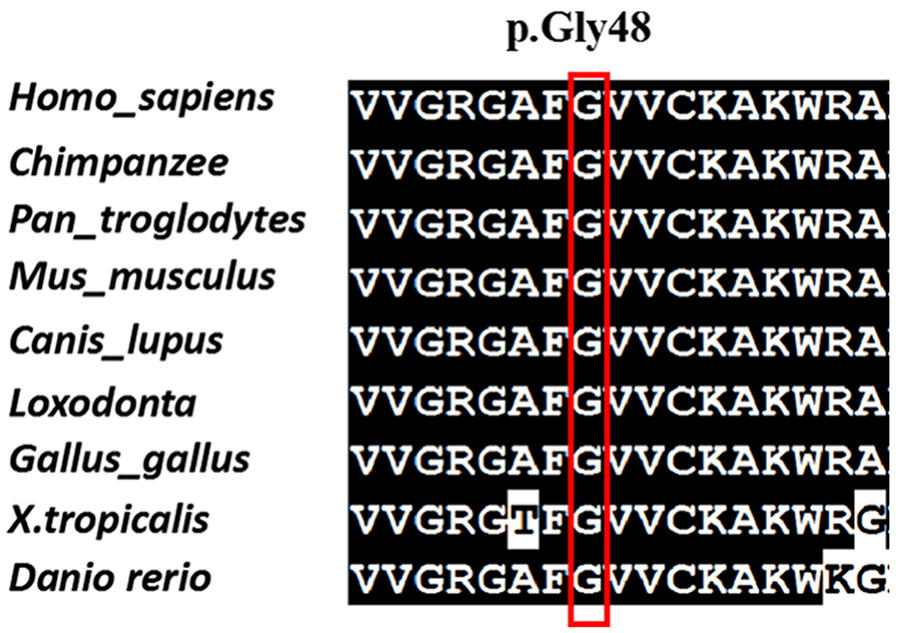
Multispecies alignment showing the strong conservation of MAP3K7 p.Gly48.

**Figure 3:**
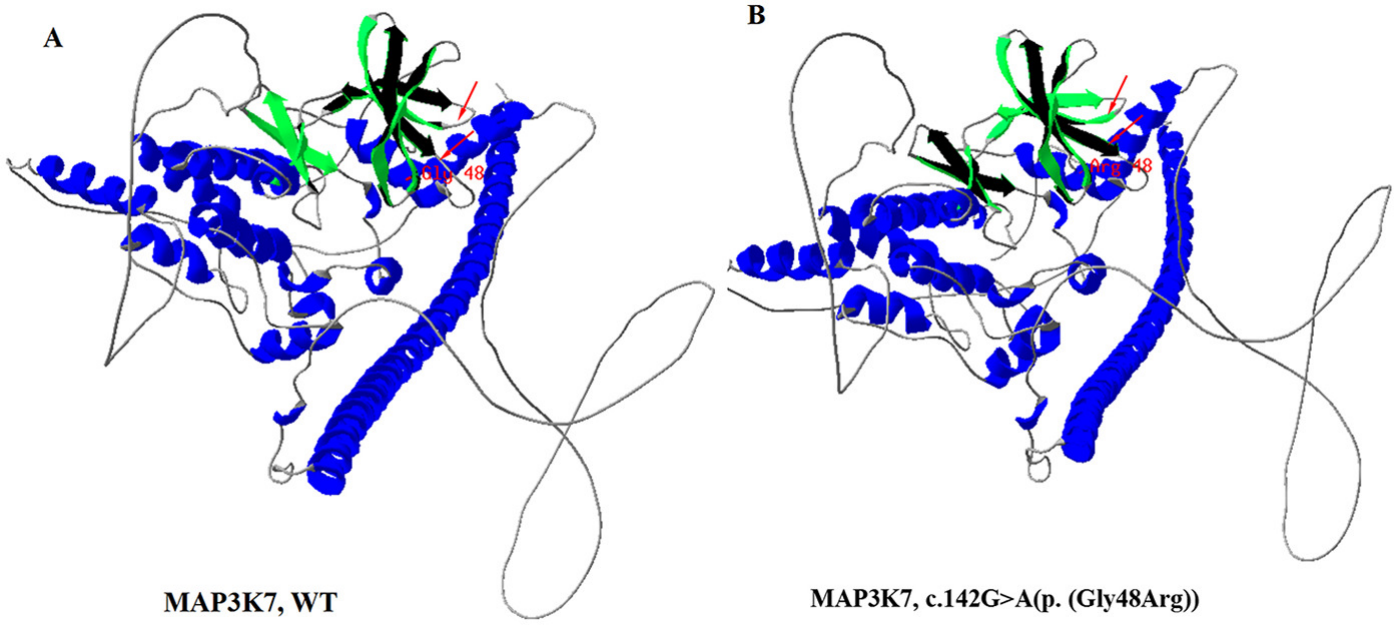
Three-dimensional structures of MAP3K7 protein. **(A)** Wild-type, **(B)** c.142G > A[p. (Gly48Arg)] mutant-type. Three-dimensional structure modelling predicted an increase in the proportion of β-sheet regions, and a decrease in random coil regions in the mutant protein. The dimer alterations are indicated by an arrow.

The variant c.142G > A[p. (Gly48Arg)] was not found in any other member of the family, confirming that it is *de novo*. In addition, the biological parent-child relationship was confirmed by short-tandem-repeat (STR) analysis, which verified that the patient is the biological child of the tested parents. To the best of our knowledge, this variant has not been reported before and isn't present in public databases, such as the Exome Sequencing Project, gnomAD, 1,000 Genomes Project, Single Nucleotide Polymorphism Database, and disease-related databases like ClinVar and the Human Gene Mutation Database. A multiple sequence alignment revealed that the sequence at residue Gly48 is highly conserved across a wide range of organisms (Figure 2). The missense variant was predicted to have a deleterious effect on MAP3K7 by multiple in silico prediction tools, including SIFT, PolyPhen2 and CADD. Three-dimensional modelling of wild-type (WT) and variant protein sequences suggests that the additional arginine gained by the MAP3K7-Gly48Arg variant alters the secondary and tertiary structures by increasing local β-folding, this change may affect structural stability (Figure 3). Variants reported to date to be associated with CSCFS are all located in the kinase domain of TAK1. The Gly48Arg found in this case, like other known variants, is located in the kinase structural domain of MAP3K7. *In vitro* experiments have shown that a variant at the same amino acid residue (Gly48Glu) decreases TAK1 autophosphorylation and disrupts TAK1-dependent signalling pathways, resulting in congenital abnormalities (14). Therefore, we propose that the new variant functions in a manner similar to the p.Gly48Glu mutation. It affects protein expression and downstream signaling pathways, resulting in multiple congenital anomalies in patients. The c.142G > A[p. (Gly48Arg)] was classified as likely pathogenic (PS2, PM2, PM5, PP3) according to the AMP/ACMG guidelines for interpretation of sequence variant (18) (Table 1). This finding confirms that the *MAP3K7* variant was likely to be responsible for the neurodevelopmental disorders in this family.

To date, only 27 affected individuals (including our patient) from 24 unrelated different families have been reported with variants in *MAP3K7* (8, 10, 12–17). An overview of the clinical presentation of CSCFS patients previously reported in the literature is provided in Table 2. Some common clinical features are observed in this disease. All patients had dysmorphic facial features, although these were variable to some extent. Among the most common dysmorphic features were hypotonic face (6/14), full cheeks (20/24), low-set ears (14/21), posteriorly rotated ears (16/23), hypertelorism (17/24), strabismus (13/21), ptosis (9/21), upslanting palpebral fissures (13/21), epicanthal folds (7/12), periorbital fullness (13/20), anteverted nares (18/23), round tipped nose (10/14), smooth/long philtrum (18/20), and high arched palate (5/8). Congenital heart defects were observed in most patients (19/25), including common atrial septal defects (8/20), valvular dysplasia (13/23), cardiomyopathy (6/16)) and other cardiac malformations. Interestingly, most of the congenital heart defects in these patients were left-sided, ranging from valvular insufficiency to hypoplastic left heart. Skeletal and limb abnormalities including joint laxity (17/19), scoliosis (8/18), vertebral anomalies (9/13), carpal/tarsal fusion (8/13), brachydactyly (15/17), pectus excavatum (6/13), delayed bone age (6/8), and cervical spine fusion (9/10) have been observed in a majority of patients with CSCFS, and with increasing age these deformities can become more pronounced and complex. Growth retardation is another important complication in these patients. It may be partly due to feeding problems and gastrointestinal problems, 95% (19/20) of the patients had feeding difficulties in infancy and 87.5% (7/8) had gastroesophageal reflux and gastrointestinal motility disorders. 68% (17/25) of the patients showed short stature. Growth hormone deficiency also affected the patients' linear height to some extent. In studies by Le Goff et al. and AbuBakr et al., growth hormone deficiency was identified in four patients exhibiting short stature (8, 12). Hearing loss was observed in most of the patients (15/20), which could be attributed to inner ear deformity and fusion of stapes with round window, which may be improved by surgical correction. Only two patients had mild intellectual disabilities. However, autism spectrum disorder was evident in 4/9 patients, as was hypotonia (10/12) and brain abnormality (4/7).

**Table 2:** Summary of the clinical features of the patients with cardiospondylocarpofacial syndrome.

Patients clinical data	Our patient	Le Goff et al. (8)	Minatogawa et al. (13)	Morlino et al. (10)	AbuBakr et al. (12)	Woerden et al. (14)	Vasishta et al. (15)	Shepherd et al. (16)	Nyuzuki et al. (17)	Total
Patients	*n* = 1	*n* = 6	*n* = 1	*n* = 1	*n* = 1	*n* = 14	*n* = 1	*n* = 1	*n* = 1	*N* = 27
Gender	M	F:2, M:4	M	F	M	F:10, M:4	F(fetus)	M	F	F:15, M:12
Growth delay										
Short stature	+	3/6	+	+	+	9/14			+	17/25
Feeding difficulties In infancy	+	5/5	+	+	+	10/11				19/20
Failure to thrive	+	5/5	+	+	+			+	+	11/11
Neurological abnormality										
Hypotonia	+		+	+		7/9		+		11/13
Behavior Disorders	−			−	+	3/6				4/9
Brain abnormality	+ (Hemispheric asymmetry and widening of the right frontotemporal extracerebral space)		−	−	−	3/4				4/8
Speech delay	+		+		+					
Motor delay	+		+		+			+	+	
Intellectual disability	+		+							
Facial dysmorphism										
Hypotonic face	−				+	4/12				5/14
Full cheeks	+	6/6		+	+	9/13	+		+	20/24
Low-set ears	+	5/6	+	+	−	5/10			+	14/21
Posteriorly rotated ears	+	5/6		+	+	6/12			+	16/23
Hypertelorism	−	5/6		+	−	8/12	+		+	17/24
Strabismus	−	3/6		−	+	8/13				13/21
Ptosis	+	3/6		+	+	3/12				9/21
Upslanting palpebral fissures	−	5/6			+	6/11				13/21
Epicanthal folds	−			+		6/10				7/12
Periorbital fullness	+	6/6		+	−	6/11				13/20
Anteverted nares	+	5/6		+	+	7/11	+		+	18/23
Round Tipped nose	−			−	−	9/10				10/14
Smooth/ long philtrum	+	5/6		+	+	7/9	+		+	18/20
High arched palate	−				−	5/8				5/8
Micrognathia	−				−	3/12				
Webbed neck	−				−	3/10				
Wide mouth	−	3/6		+	+					
Short lingual frenulum	+									
Cardiovascular										
Congenital heart defect	+	6/6		+	+	7/13	+		+	19/25
Septal defects	+	2/6		+	+	2/10			+	8/20
Cardiomyopathy	+			+		4/14				6/16
Valve dysplasia	+	5/6		+		3/12	+		+	13/23
Pulmonary hypertension	+									
Skeletal and limb abnormalities										
Joint laxity	+	5/5		+	+	7/9		+		17/19
Scoliosis	−	2/5		+	+	2/9				8/18
Vertebral abnormalities	−	1/3		+	+	4/6			+	9/13
Carpal/tarsal fusion	−	4/4			+	2/6				8/13
Brachydactyly	+	5/5		+	+	6/8				15/17
Pectus excavatum	−			+	+	3/9				6/13
Delayed bone age	+	3/5			+					6/8
Cervical vertebral fusion	−	5/5		+	+				+	9/10
Ear, Nose, and Throat										
Hearing loss	+	5/5		+	+	5/10			+	15/20
Recurrent otitis	+	3/3		−	+			+		7/8
Inner ear malformation	−	5/5			+					6/7
Gastrointestinal Findings										
Gastroesophageal reflux	−	4/5		+	+			+		7/8
Gastrostomy tube	−	2/5		+	+					4/7
Gastrointestinal dysmotility	+ (Intestinal obstruction and intussusception)				+					
**Abnormal skin texture/scarring**	+			+	+					
**Abnormal genitalia**	+	2/6		-	+	2/14				

Clinically, our patients presented with a similar profile to those reported in previous cases, including short stature, hypotonia, feeding difficulties, facial dysmorphism, congenital heart defect, and skeletal and limb abnormalities. However, we still found some previously unreported features which extend the phenotype associated with CSCFS. Our patients exhibited mild intellectual disability, delayed language and motor development. Interestingly, intellectual disability was described mainly in patients with FMD2 and in only one case of CSCFS (7, 13). In addition, we observed hypotonia in many patients. Although this condition improved with age, delayed motor development in infancy cannot be ruled out in these individuals. The short lingual frenulum, first observed in our patients, may also be a factor affecting speech development. Most patients experienced growth issues, largely attributed to feeding difficulties and gastrointestinal problems, with the majority undergoing gastrostomy. Our patients also showed growth retardation due to feeding difficulties and gastrointestinal issues. However, intestinal obstruction and intussusception observed in our patients were not reported in others. Furthermore, compared to previously reported cases, our patient exhibited milder skeletal and limb abnormalities characterised by joint laxity, brachydactyly and delayed bone age. Finally, although variable brain anomalies have been observed in CSCFS patients, asymmetrical cerebral hemispheres with widened right frontotemporal extracerebral space was observed for the first time (8). These results further highlight the complexity and heterogeneity of the CSCFS phenotype. Pathogenic variants of the *MAP3K7* gene have been reported to cause two distinct but overlapping syndromes: cardiospondylocarpofacial syndrome (CSCFS) and frontometaphyseal dysplasia type 2 (FMD2). The expressivity of these syndromes ranges from isolated valvular disease to multisystem connective tissue manifestations (7, 10). Studies have also demonstrated significant heterogeneity within and between families (7, 13, 17). Additionally, TAK1 (encoded by MAP3K7) is widely expressed in various tissues, including the embryonic cranial neural crest, enteric mesenchyme, and dorsal root ganglia. There, it orchestrates the NF-*κ*B, p38 and JNK signalling pathways, which are critical for craniofacial midline formation, gut motility, and tongue morphogenesis (21–23). Therefore, it cannot be ruled out that the *MAP3K7* variant causes developmental abnormalities in these organs. Furthermore, *MAP3K7* variants in patients with CSCFS are characterised by loss of function (LOF) (7). The phenotypic variations observed in these patients may stem from loss-of-function mutations occurring at different sites. To assess the significance of these differences, further functional studies on more patients and *MAP3K7* variants are needed.

In summary, we uncovered a novel *de novo* heterozygous missense variant in the *MAP3K7* gene in a Chinese boy with CSCFS. This is the first report to describe a Chinese family with a *MAP3K7* variant. The variant was associated with developmental delay, short stature, hypotonia, feeding difficulties, facial dysmorphism, congenital heart defect, skeletal and limb abnormalities, gastrointestinal issues, and brain abnormalities.These data further extend the spectrum of variation and phenotypes in CSCFS.

## Data Availability

The original contributions presented in the study are included in the article, further inquiries can be directed to the corresponding author.
